# GWAS Mediated Elucidation of Heterosis for Metric Traits in Cotton (*Gossypium hirsutum* L.) Across Multiple Environments

**DOI:** 10.3389/fpls.2021.565552

**Published:** 2021-05-20

**Authors:** Zareen Sarfraz, Muhammad Shahid Iqbal, Xiaoli Geng, Muhammad Sajid Iqbal, Mian Faisal Nazir, Haris Ahmed, Shoupu He, Yinhua Jia, Zhaoe Pan, Gaofei Sun, Saghir Ahmad, Qinglian Wang, Hongde Qin, Jinhai Liu, Hui Liu, Jun Yang, Zhiying Ma, Dongyong Xu, Jinlong Yang, Jinbiao Zhang, Zhikun Li, Zhongmin Cai, Xuelin Zhang, Xin Zhang, Aifen Huang, Xianda Yi, Guanyin Zhou, Lin Li, Haiyong Zhu, Baoyin Pang, Liru Wang, Junling Sun, Xiongming Du

**Affiliations:** ^1^State Key Laboratory of Cotton Biology/Institute of Cotton Research, Chinese Academy of Agricultural Sciences (ICR, CAAS), Anyang, China; ^2^Cotton Research Institute, Ayub Agricultural Research Institute, Multan, Pakistan; ^3^Anyang Institute of Technology, Anyang, China; ^4^Henan Institute of Science and Technology, Xinxiang, China; ^5^Cash Crops Research Institute, Hubei Academy of Agricultural Sciences, Wuhan, China; ^6^Zhongmian Seed Technologies Co., Ltd., Zhengzhou, China; ^7^Jing Hua Seed Industry Technologies Inc., Jingzhou, China; ^8^Cotton Research Institute of Jiangxi Province, Jiujiang, China; ^9^Key Laboratory for Crop Germplasm Resources of Hebei, Agricultural University of Hebei, Baoding, China; ^10^Guoxin Rural Technical Service Association, Hebei, China; ^11^Zhongli Company of Shandong, Shandong, China; ^12^Hunan Cotton Research Institute, Changde, China; ^13^Sanyi Seed Industry of Changde in Hunan Inc., Changde, China

**Keywords:** GWAS, heterosis, F_1_ hybrid, hQTNs, multiple environments, upland cotton

## Abstract

For about a century, plant breeding has widely exploited the heterosis phenomenon–often considered as hybrid vigor–to increase agricultural productivity. The ensuing F_1_ hybrids can substantially outperform their progenitors due to heterozygous combinations that mitigate deleterious mutations occurring in each genome. However, only fragmented knowledge is available concerning the underlying genes and processes that foster heterosis. Although cotton is among the highly valued crops, its improvement programs that involve the exploitation of heterosis are still limited in terms of significant accomplishments to make it broadly applicable in different agro-ecological zones. Here, F_1_ hybrids were derived from mating a diverse Upland Cotton germplasm with commercially valuable cultivars in the Line × Tester fashion and evaluated across multiple environments for 10 measurable traits. These traits were dissected into five different heterosis types and specific combining ability (SCA). Subsequent genome-wide predictions along-with association analyses uncovered a set of 298 highly significant key single nucleotide polymorphisms (SNPs)/Quantitative Trait Nucleotides (QTNs) and 271 heterotic Quantitative Trait Nucleotides (hQTNs) related to agronomic and fiber quality traits. The integration of a genome wide association study with RNA-sequence analysis yielded 275 candidate genes in the vicinity of key SNPs/QTNs. Fiber micronaire (MIC) and lint percentage (LP) had the maximum number of associated genes, i.e., each with 45 related to QTNs/hQTNs. A total of 54 putative candidate genes were identified in association with HETEROSIS of quoted traits. The novel players in the heterosis mechanism highlighted in this study may prove to be scientifically and biologically important for cotton biologists, and for those breeders engaged in cotton fiber and yield improvement programs.

## Introduction

The phenomenon of biological progeny outperforming either of their parents is defined as heterosis (Shull, 1922). The concept of heterosis dates back to early experiments on inbreeding and its complementing hybrid vigor (Shull, 1908, 1909). Generally, heterosis is assumed to be highly characteristic of allogamous crops but less common in purely autogamous crops for improvements in their total growth rate, fitness, and biomass production, as well as yield (Lippman and Zamir, 2007; Chen, 2013; Schnable and Springer, 2013).

Highly conceptual quantitative genetic models attributed to heterosis, known as dominance (Xiao et al., 1995), over dominance (Li et al., 2001, 2008) and epistasis (Yu et al., 1997), are considered insufficient for explaining its basic molecular mechanism. Currently, many omics studies are trying to describe changes in gene expression across genome and histone modifications, deoxyribonucleic acid (DNA) methylation, and micro RNAs. These aspects are being studied in hybrids and their parents as well, but nevertheless the genetic mechanism underlying this phenomenon remains elusive (He et al., 2010, 2013; Fujimoto et al., 2012; Groszmann et al., 2015; Miller et al., 2015). With the revolution in computational methods and extensive advancements in genome sequencing methods, deployment of genome-wide association studies (GWAS) has proven to be a tremendously powerful tool. It has been applied especially for exploring the specific genetic loci potentially accountable for heterotic traits in crop plants (Atwell et al., 2010; Kump et al., 2011; Huang et al., 2012; Meijón et al., 2014).

Cotton is widely cultivated across the globe as a natural fiber crop on a commercial basis. In this respect, *Gossypium hirsutum* is responsible for approximately 95% of cotton production worldwide (Grover et al., 2015). China is considered a top cotton-growing territory given the vast number of genotypic diversity and agro-ecological zones for cotton that exist in the country. Although both wider adaptability and increased productivity attributes are associated with Upland Cotton crop, the low quality of its fiber product requires novel improvements and advances in spinning technology. According to previous investigation, we know now that a substantial amount of heterosis exists in cotton (Sarfraz et al., 2018). Both India and China are enjoying substantial benefits of hybrid cotton via a cryptic process of heterosis since the last century (Basunanda et al., 2010). Nowadays, the focus of most studies in top edible crops like maize (Frascaroli et al., 2007), rapeseed (Radoev et al., 2008), and rice (Xiao et al., 1995) is on the heterosis mechanism. Linkage mapping studies that utilized segregating populations of these crops have found more than a single gene involved and related mechanisms for hybrid vigor existing among them (Schnable and Springer, 2013). Furthermore, the underlying genetic basis of heterosis in maize and rice is somewhat different. The prominent peculiarity related to the fact seems to be highly correlated to their self-pollinated or open-pollinated nature (Garcia et al., 2008). There is now a need to also study this confounding process systemically in Upland Cotton.

The use of bi-parental crossing scheme yields little additional information, but GWAS or linkage disequilibrium (LD) mapping have emerged as extensively utilized, powerful techniques for the genetic dissection of complex mechanisms via high density molecular markers (Zhu et al., 2008). Further, single nucleotide polymorphism (SNP) assays have empowered GWAS for such studies, especially as related to intricate cotton traits. Yet only a few reports of GWAS mapping have used F_1_ populations based on SNP markers in cotton.

Accordingly, this study was planned and executed in which GWAS was used to detect allelic variation in the genome of cotton and identify candidate SNPs strongly associated with economic quantitative traits. Restriction-site associated DNA (RAD) sequencing was applied to 1136 F_1_ individuals of upland cotton; these were then evaluated phenotypically in 10 environments over 2 years. The current study aimed at the (i) detection of SNP loci associated with trait performance of F_1_ hybrids and of Quantitative Trait Nucleotides (QTNs) related to the heterosis of these traits and (ii) the identification and validation of potential candidate genes, especially heterotic Quantitative Trait Nucleotide (hQTNs), related to the investigated traits.

## Materials and Methods

A set of 284 diverse Upland Cotton accessions, collected from gene bank of ICR, CAAS, Anyang, Henan, along with four highly ranked and renowned commercial cultivars. This collection is from a range of different regions of China. A major portion, 83.3% (240), was collected from various conventional cotton-growing areas, including the Yangtze River region, the Yellow River Valley, and Northern and Southern areas of China. The other 16.67% (48) consisted of introductions from different geographical areas of United States, Ivory Coast, Australia, Russia, Turkmenistan, Uganda, Kenya, Burundi, Chad, Sudan, and Vietnam (11 different countries), as shown in Supplementary Table 1. The subgroups present in the experimental accessions were estimated using ADMIXTURE software. This panel of accessions was designated as male and female parents based on their performance and commercial value.

Line × Tester mating design was proposed for the current study to obtain different variables related to heterosis and combining abilities, for the purpose of utilizing them as variables in association studies. For this purpose, 284 female parents (lines) were crossed with four testers as males, namely 7886, A971Bt, 4133Bt, SGK9708 coded, respectively as tester A, C, D, and E to produce the F_1_ hybrid populations. The ensuing F_1_ hybrids were divided into four groups (SetA, SetC, SetD, and SetE) according to their respective parents.

Each set of F_1_ hybrids along with their respective parents were subjected to a field evaluation for plant phenotyping. Field plantations of the experimental material were established during the crop seasons of 2012 and 2013 at 12 locations in cotton-growing belts of China, these mainly spanning parts of the Yangtze River region (Changsha, Changde, Jiujiang, Hefei, Wuhan, and Jingzhou) and the Yellow River region (Anyang [ICR], Anyang [Beibi], Hejian, Dongying, Baoding, and Xinxiang). These locations were selected on the basis of significant differences in agro-ecological features including climate, amount of precipitation, temperature, soil fertility, growing period, and cultural practices.

Field experiments were carried out that used a triplicated randomized complete blocked design (RCBD) at all 12 locations. A single row of genotypes was 8.0 m, row × row distance was maintained at 0.8 m the plant × plant distance was kept at 0.3 m in the Yellow River region and at 0.5 m in the Yangtze River region. The distance between replications was 1.0 m. Local cotton growing practices were followed for sowing; i.e., direct sowing of delinted seeds/seeds with lint or transplanting of seedlings using the method predominantly used in that given region. All recommended agronomic practices–fertilizer application, seed treatment, seed rate, sowing methods, thinning, cultural practices, irrigation, insect pest control and weed management–were followed in similar manner to establish and maintain a good crop stand on all 12 experimental locations.

Data collection for all traits under study was carried out for all experimental units, by following the unanimous standard Descriptors for Data collection used for Cotton Germplasm, which was developed based on guidelines issued by the International Plant Genetic Resources Institute (IPGR). Ten individual guarded plants were randomly selected and tagged for data collection related to agronomic traits as well as quality-related characters. When the crop had about 70% open bolls, 30 bolls from each tagged individual plant (middle branches) per plot were harvested and examined for agronomic and fiber quality traits. The ginning of collected seed cotton samples was done using a roller-gin. About 150 g of ginned and clean lint samples were taken and sent to the Laboratory of Quality and Safety Risk Assessment for Cotton Products, Anyang, Henan, China, to examine fiber quality-related traits. Fiber quality analysis was carried out using high-volume instrument (HVI). Ten phenotypic traits–boll weight (BW), lint percentage (LP), fiber fineness or micronaire (MIC), fiber strength (FS), fiber length (FL), fiber elongation (FE), fiber uniformity (FU), fiber uniformity index (FUI) number of bolls per plant (BN) and plant height (PH)–were recorded (Supplementary Table 2) from 284 individuals of the above-mentioned F_1_ populations, as well as their parents, planted at different locations as experimental units during the two different years.

### Sample Preparation for RAD Sequencing

Young fresh leaves were collected from each genotype and immediately frozen and then stored at −80°C. Genomic DNA was extracted following the CTAB method (Paterson et al., 1993) albeit with some modifications. The purified DNA was digested with FastDigest *TaqI* (Thermo Scientific Fermentas, United States), at 65°C for 10 min. Bar-coded adapters were ligated to the digested DNA fragments with T4 DNA ligase (Enzymatics, United States), during 1 h incubation at 22°C. The samples were then heated at 65°C for 20 min, after which 24 samples were pooled. The DNA fragments (400–600 bp) were purified from 2% agarose gel electrophoresis with the help of the QIA-quick Gel Extraction kit (QIA, Qiagen, Valencia, CA, United States). Adapter-ligated DNA fragments were further amplified by polymerase chain reaction (PCR), using the Phusion-High-fidelity DNA-polymerase (Finnzymes, Thermo Scientific, United States). Next, these amplified fragments were separated via agarose gel electrophoresis, and the ensuing DNA fragments (400–600 bp) were purified with the QIA-quick PCR Purification kit (Qiagen, Germany). Finally, the purified libraries were quantified on a 2100 Bioanalyzer (Agilent, United States) and each library sequenced by the Hi-Seq 2000 system (Illumina, United States). The raw reads were then aligned with the *G. hirsutum* L. TM-1 reference genome (v.1.1)^1^, using the “mem -t 8” parameter of the BWA program (Zhang et al., 2015). The GATK and SAMTools packages were used for SNP calling, after which any SNPs with a high missing-data rate (>40%) and a low minor allele frequency (MAF) (<5%) were eliminated (Li et al., 2009; McKenna et al., 2010). The generated sequencing data have been deposited into the NCBI database (accession number: PRJNA353524).

### Phenotypic Data Analysis

The collected data for 10 agronomic and fiber quality traits (BW, LP, MIC, FS, FL, FE, FU, FUI, BN, and PH) recorded from 284 F_1_ crosses were subjected to univariate analysis for determining variability among the studied traits (Gomez et al., 1984). The relative increase or decrease (in percentage) for F_1_ hybrids over their respective parental values was determined for the estimation of possible heterotic effects for the agronomic and quality traits, by using these formulas (Fehr, 1987):

Heterobeltosis was calculated this way:

$$\text{HB}=\frac{\,\left(\text{F}\,1-\text{HP}\right)}{\text{HP}}\,\text{x}\,100\%$$

Mid-parent heterosis (MP) was calculated this way:

$$\text{MP}=\frac{\mathrm{F1}-\text{P}1+\mathrm{P2})/2}{\text{P}1+\mathrm{P2})/2}\,\text{x}\,100$$

Competitive heterosis (K) over local cultivar(s)/Check(s) (CK) was calculated as:

$$\text{K}=\frac{\,\left(\text{F}\,1-\text{CK}\right)}{\text{CK}}\,\text{x}\,100$$

In the current study, two standard heterosis measures, K3 and K4, were calculated by using two commercial Chinese cotton cultivars, i.e., Rui za 816 and Eza mian 10 hao (Tai D5), respectively.

The heterosis index (HI) was calculated as follows:

$$\text{HI}=\frac{\mathrm{F1}}{\text{P}1+\text{P}2)/2}\,\text{x}\,100$$

The specific combining ability (SCA) variance was calculated by using Line × Tester variance analysis (Singh and Chaudhary, 1977).

### Genotypic Data Analysis

To explore different genetic factors presumably associated with heterosis in cotton, GWAS was performed by considering familial relatedness as well as population structure (Yu et al., 2006), utilizing the data for all traits under investigation for 2 years at 10 locations. The experimental genotypes were examined using “Restriction-site associated DNA (RAD) sequencing.” The BWA v.0.7.12 software was utilized to analyze all the SNP data. Only the reads mapped uniquely to the reference genome and the SNPs with high missing rate (>40%) and MAF (<5%), were considered for elimination for conducting GWAS.

The paired-end reads of each individual were identified by its barcode and aligned against the reference genome, using the BWA v.0.7.12 (Staples et al., 2014). The program SAMTools v.0.1.18 (Price et al., 2006) was used to generate the consensus sequences for every individual under study and further preparation of input data for SNP calling; the latter was carried out by realSFS v0.983^2^, using Bayesian-based estimation. The data obtained from the four sets of F_1_ genotypes along with parents were considered for calculations, based on principle that the 284 female parents and 4 male parents must be homozygous; otherwise, they were removed. Moreover, only those female and male parents having a different genotype (e.g., AA, BB) were considered for analysis, to ensure a heterozygous F_1_ genotype. The expected F_1_ genotype was calculated, by focusing on the genotype of respective male and female parent and heterozygous SNPs in either of the parents have been scored as missing.

Single nucleotide polymorphisms that met the following criteria were removed: (1) Length (distance) between two adjacent SNP loci was less than 5 bp. (2) SNPs with call rates lower than 70% (Wright et al., 2019) in the whole population. (3) A MAF < 0.05. (4) The proportion of its heterozygous genotypes was above 20%. Here, four F_1_ sets: F_1__A, F_1__C, F_1__D and F_1__E, were finalized for the GWAS analysis using high-quality SNPs.

The GWAS analysis was performed on filtered high-quality SNPs, using EMMAX software, by following an efficient mixed-model association-expedited model designation, as described by Kang et al. (2010), for which a threshold of *p* = 1.0 × 10^–5^ was used throughout. For the visualization of results, Manhattan and quantile–quantile plots were constructed in R using the package “qqman.” The peak SNPs with the highest *p*-value as well as their detection across multiple environments, were considered as key SNPs. For further confirmation, the favorable allelic variations of the key SNPs were identified for each trait variable (trait phenotype, SCA, and heterosis types). Box plots for the relative phenotypic values were drawn in R software. The HAPLOVIEW 4.2 software (Zhang and Endrizzi, 2015) was used to carry out the haploblock analysis. All genomic positions provided here are based on the *G. hirsutum* L. reference genome (v.1.1) (Zhang et al., 2015).

Gene ontology (GO) analysis was performed using the cotton functional genomics database^3^, to propose annotated putative candidate genes for each locus. For the transcriptome-based predictions, the gene expression database (TM-1) (Zhang et al., 2015) was used for the assessment of specific expression patterns of these nearby genes across various tissues: an organ or perhaps different growth and development stages of cotton viz. root, leaf, stem, torus, petal, stamen, pistil, and fiber (5 DPA to 25 DPA) and ovule (−3 DPA to 35 DPA). By applying the above-mentioned criteria within a 100-kb flanking window, candidate genes were thus selected. The differential expression patterns of these genes (i.e., those with expression level >1) were plotted in a heatmap.

## Results

### Phenotypic Characteristics Evaluation

**FIGURE 1:**
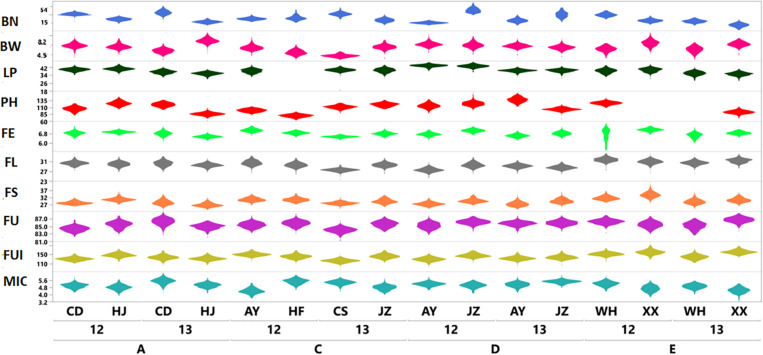
Violin plots based on phenotypic variation of ten agronomic and fiber quality traits of F_1_ hybrids (*Y*axis) from four sets (SetA, SetC, SetD, and SetE) across multiple environments for two years; 2012 and 2013 (*X*axis). Legends on top right in different colors are representing ten evaluated phenotypic traits.

**FIGURE 2:**
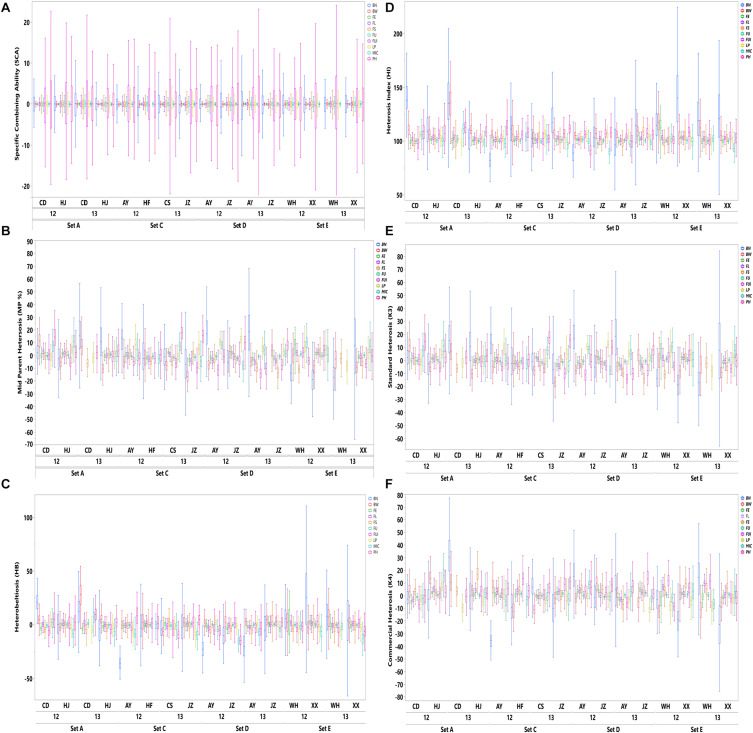
Distribution of SCA and five heterosis types (HB, MP, HI, K3, and K4) of agronomic and fiber quality traits among four F_1_ hybrid sets (SetA, SetC, SetD, and SetE) across multiple environments for years 2012 and 2013. Legends on the top right in different colors are depicting ten evaluated phenotypic traits.

The results shown in Figure 1 revealed variation among the different agronomic and fiber quality traits performance for F_1_s and parents. Upper and lower ends as shown in the vertical projections are assumed to represent highest to lowest data points (further details in Supplementary Table 3). Figure 2 shows the five types of studied heterosis related to fiber quality and agronomic traits performance, along with SCA among four sets of crosses (284 each). The averaged MP heterosis of each trait showed a positive trend with highest range occurring for BN (251.8) and the lowest for FU (11.3). A considerable range of variation was observed regarding these variables, thus providing sufficient ground for their further GWAS (Supplementary Table 3).

### Population Structure

**FIGURE 3:**
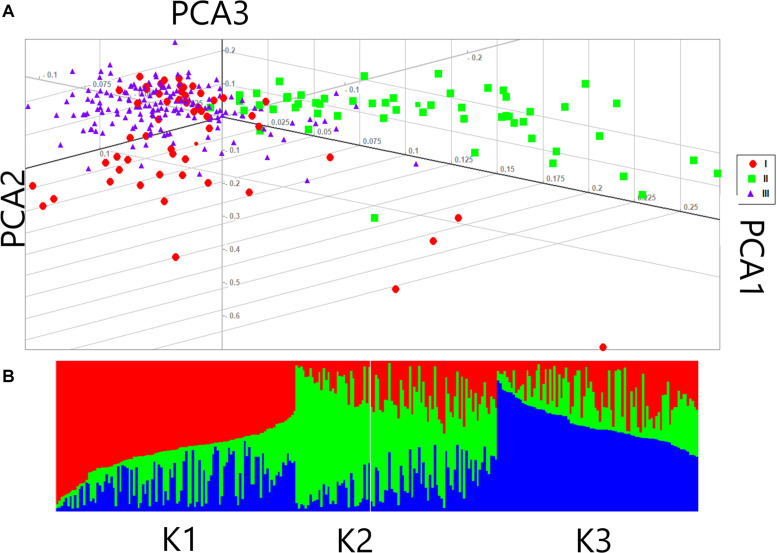
Population structure of 284 female parents in the association panel **(A)** Principal component analysis (PCA) of female lines **(B)** Population structure of 284 female parents (*K* = 3).

Based on the fact that population structure increases the authenticity of identified SNPs, the number of subgroups that existed in the experimental accessions was critically estimated. The experimental accessions encompassed subgroups on the basis of their different geographic origins. The results from ADMXTURE software analysis of the experimental accessions could be divided into three divergent groups: Group I, II, and III with 86, 64, and 134 individuals, respectively (Figure 3). A genotypic principal component analysis (G-PCA) was performed in EIGENSOFT v. 6.0.1 software; this clearly displayed the top three eigen vectors: PC1, PC2, and PC3 (Figure 3). Both analyses clearly distinguished the accessions into three groups on the basis of which further GWA studies were implemented, with *Q* = 3.

### Genome Variation Based on the SNPs

**FIGURE 4:**
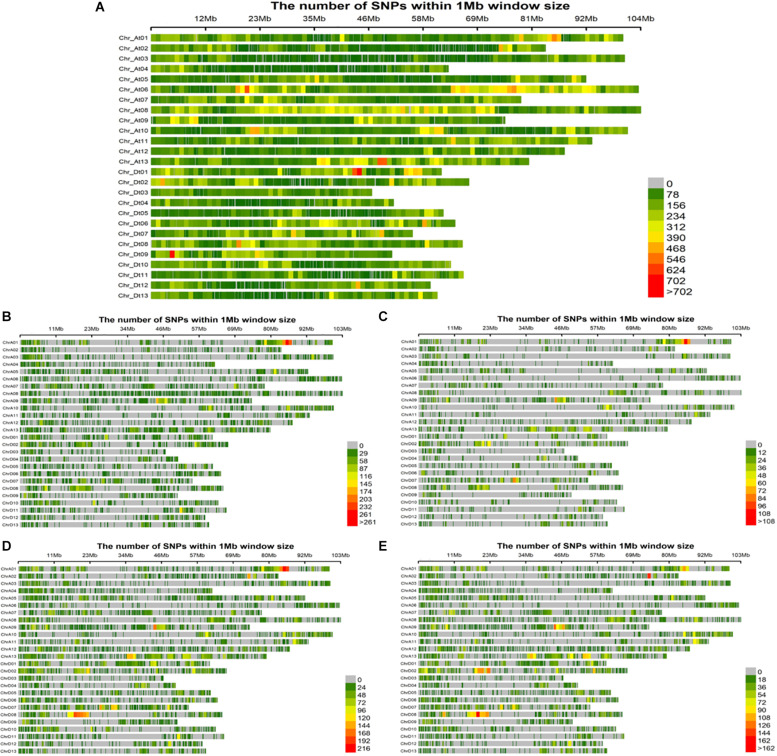
Single nucleotide polymorphism (SNP) distributions on 26 chromosomes of **(A)** parents, **(B)** F_1__A, **(C)** F_1__C, **(D)** F_1__D, and **(E)** F_1__E. A_*t*_1∼A_*t*_13 and D_*t*_1∼D_*t*_13 in vertical axis are the serial number of 26 chromosomes; the horizontal axis shows chromosome length (Mb); = 0 ∼>702 depicts SNP density (the number of SNPs per window).

Evidently these SNPs, which totaled 252,110 in number, were not evenly distributed across entire cotton genome (A_*t*_: 151,104 and D_*t*_: 101,006). The A_*t*_ sub-genome housed a greater number of SNPs associated with the fiber quality-related traits, while the D_*t*_ sub-genome harbored more SNPs for the agronomic traits. The A_*t*_08 chromosome had the most SNPs (20,960), whereas the A_*t*_04 chromosome had the least (4,726) (Supplementary Table 4). All these SNPs were utilized for the GWAS of female parents, amounting to 35,769 high quality SNPs for the four sets of F_1_s, as follows: 18,391 SNPs for F_1__A, 7458 SNPs for F_1__C, 23,128 SNPs for F_1__D and 17,692 SNPs for F_1__E (Figures 4A–E). On chromosome A_*t*_08, maximum number of associated SNPs were found i.e., 113, while the minimum number of associated SNPs was estimated to be 16, on chromosome D_*t*_04 (Supplementary Table 5).

### SNPs’ Associations in F_1_ Sets and Heterosis Types

**FIGURE 5:**
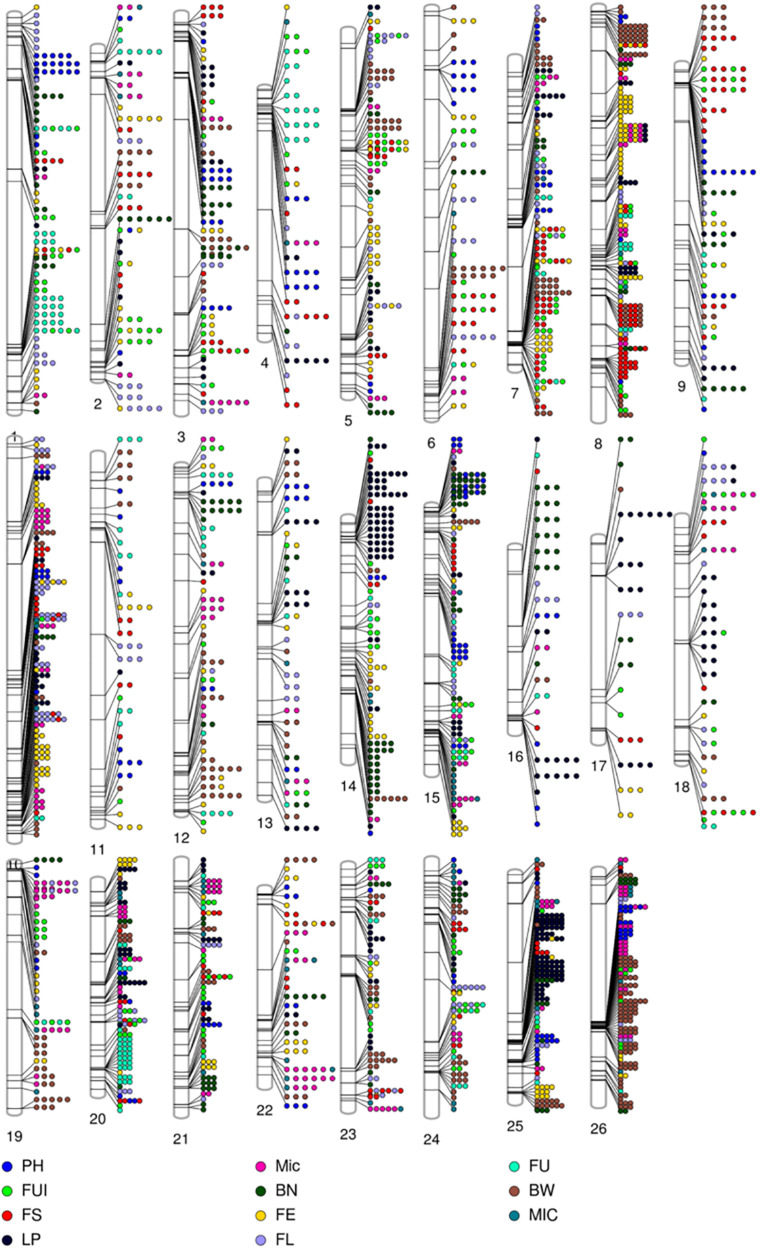
Phenogram displaying the 2847 significant (–log (*p*) ≥ 4) associations among phenotypic traits and 1348 significant SNPs residing on 26 chromosomes of upland cotton.

**FIGURE 6:**
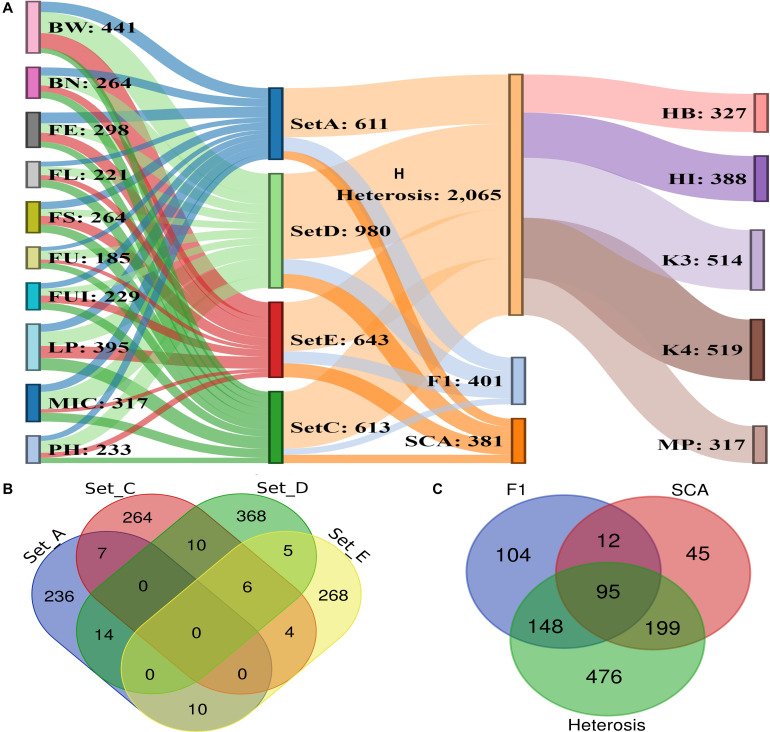
Summary of significant association signals and significant SNPs. **(A)** Representation of significant associations among 10 phenotypic traits, four F_1_ sets, five heterosis types, SCA and significant SNPs **(B)** details of significant SNPs commonly associated across four different sets of F_1_ hybrids **(C)** number of significant SNPs/hQTNs associated with heterosis types, SCA and F_1_ sets.

**FIGURE 7:**
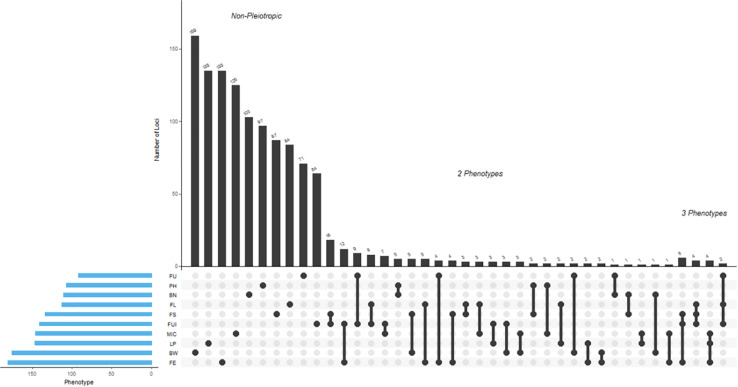
Depicted here are results from the multivariate analysis of pleiotropy. For each associated SNP, the method returns the best-fitting solution of which phenotypes were associated with that SNP. All SNPs with one or more associated phenotypes are shown here. For example, every SNP associated with FE was found to be pleiotropic for other phenotypes. The total number of pleiotropic as well as unique associated SNPs for each trait from these analyses were 181 (FE), 176 (BW), 147 (LP), 146 (MIC), 141 (FUI), 134 (FS), 113 (FL), 111 (BN), 107 (PH), and 92 (FU).

A total of 1,192 significant SNPs revealed 2,847 significant associations (−log_10_ (*p*) ≥ 4) with the 10 studied traits of the cotton parents and 4 F_1_ sets (Figure 5). The maximum number of associations was discovered for BW (441) and the minimum for FU (185). However, FE ranked highest in terms of number of associated SNPs, with 181, this being lowest for FU with 92 (Figure 6A and Supplementary Table 6). Collectively, 236, 264, 368 and 268 SNPs were revealed by F_1__A, F_1__C, F_1__D, and F_1__E sets, respectively. Furthermore, we discovered six SNPs shared in common by these three sets: F_1__C, F_1__D, and F_1__E. Seven common SNPs were found between the F_1__A and F_1__C sets, 14 SNPs were commonly shared by F_1__A and F_1__D sets, and 10 SNPs were commonly shared between the F_1__A and F_1__E sets. Similarly, 10 SNPs were observed in common between the F_1__C and F_1__D sets, four in the F_1__C and F_1__E sets, and five in the F_1__D and F_1__E sets (Figure 6B and Supplementary Table 7). However, 476 SNPs/hQTNs were found associated with the five evaluated types of heterosis. Of those, 199, 148, and 95 SNPs/hQTNs of heterosis types were also commonly shared by SCA, F_1_ sets and both SCA and F_1_ sets, respectively (Figure 6C and Supplementary Table 8). Likewise, the numbers of significant pleiotropic SNPs related to agronomic and fiber quality traits were tallied to gain insight into pleiotropy. Those details are presented in Figure 7 and Supplementary Table 9.

### Mining of Associated Key SNPs

**FIGURE 8:**
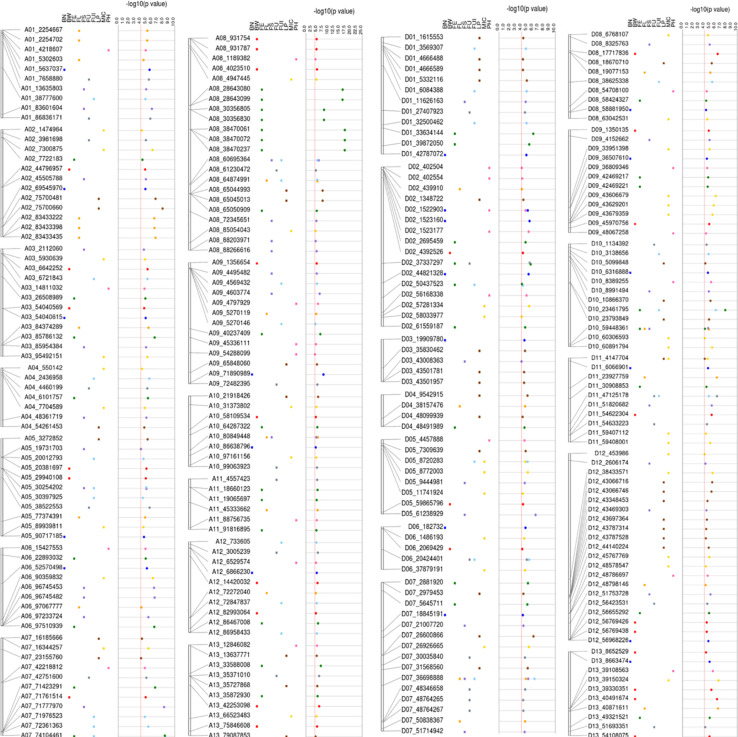
Detailed view of significant SNPs related to five types of heterosis, F_1_, SCA and studied traits on 26 chromosomes with their physical positions (bp).

A total of 298 significant (−log_10_ (*p*) > 4) key SNPs/QTNs were identified, based on the highest *p*-value, presence in multiple environments and function i.e., boxplots and haploblock analysis (Supplementary Table 10). Figure 8 summarizes the results for the simultaneous identification of key SNPs/QTNs in different F_1_ sets. Of 298 significant key SNPs/QTNs, 271 heterotic SNPs/hQTNs were related specifically to the heterosis evaluated in this study (Supplementary Table 11). The F_1__D set contributed the highest number of key SNPs/hQTNs, with 87, followed by F_1__E set with 77 SNPs/hQTNs, the F_1__A with 59 SNPs/hQTNs and the F_1__C with 56 SNPs/hQTNs (Supplementary Table 12). A total of 19 highly stable hQTNs were detected on the basis of their simultaneous contribution by multiple paternal sources and their detection of association signals in multiple environments (Supplementary Table 13). Further investigations revealed that 8, 4, 4, 2, and 1 stable hQTNs were associated with LP, BW, FS, FL, and MIC, respectively. These hQTNs were further validated by functional analysis using genotype–phenotype interaction, SNP–SNP interaction, and gene expression.

### Identification of Candidate Genes and Their Annotation

**FIGURE 9:**
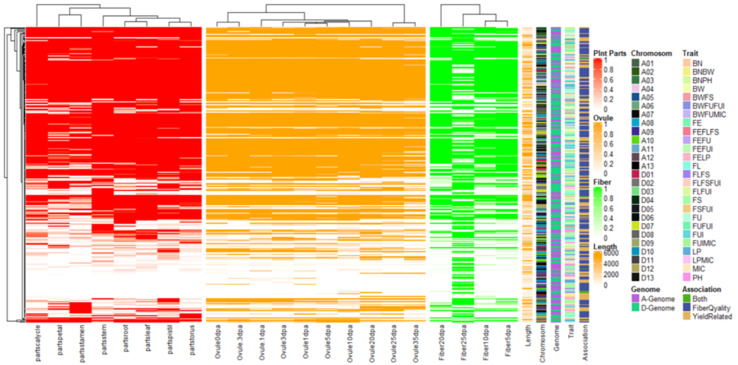
Heat map for expression patterns of the 275 genes nearby significant keys SNPs/QTNs associated with studied agronomic and fiber quality traits. Shaded portion is representing expression >1 while white portion is representing <1.

**FIGURE 10:**
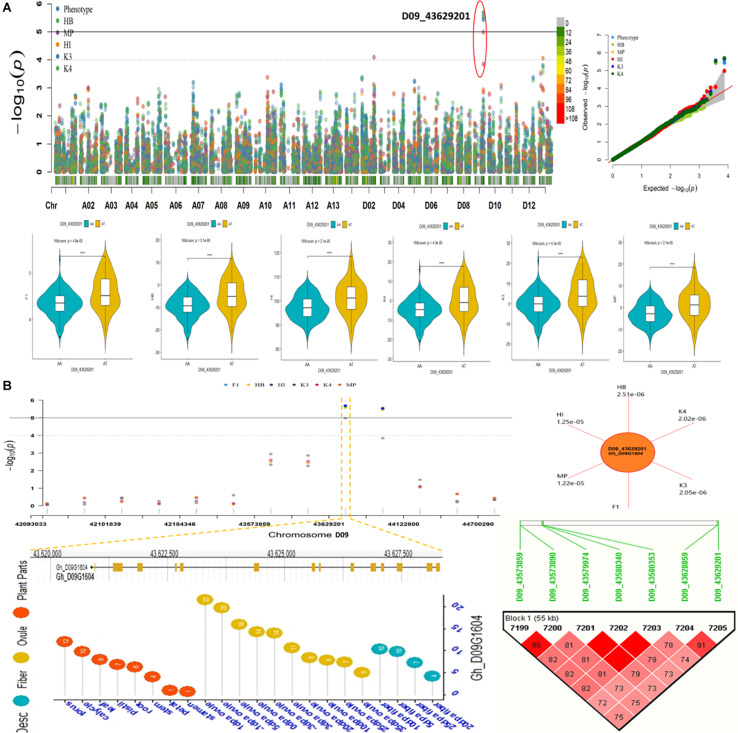
**(A)** Summary of GWAS results for Fiber micronaire (MIC) including Manhattan plots, QQ plots, violin plots displaying differences for MIC among two haplotypes of SNP/hQTN D09_43629201 in trait phenotype and five heterosis types. **(B)** Regional Manhattan plots showing presence of hQTN D09_43629201 in trait phenotype and five types of heterosis further narrowing down to genomic location of gene hqMICD09_43629201_C on chromosome D09, sun plot displaying the *p*-values of variables harboring hqMICD09_43629201_C and gene Gh_D09G1604, Expression levels of representative gene associated with MIC during different growth stages and Haplotype region (55 kb) surrounding the peak on chromosome D09 associated with MIC.

We conducted an exploration of nearby genes (i.e., 100-kb flanking window) of 298 QTNs on the basis of genes’ annotation with reference to TM-1 genome of *G. hirsutum* (Zhang et al., 2015). Overall, 275 genes (A_*t*_: 128, D_*t*_: 147) were identified for further scrutiny (Supplementary Table 11). Based on the transcriptome analysis, a heatmap of the differential expression of genes in various tissues and growth stages was plotted (Figure 9). These genes were assumed exert effects on related traits; for instance, a gene differentially expressed across fiber during the different DPA would be involved in determining agronomic quality as well as fiber quality. The GO analysis was performed using cotton functional genomics database (see text footnote 3) to annotate the putative candidate genes with biological processes, cellular components, and molecular functions (Table 1). The GO analysis revealed that those candidate genes with known functions were involved in different catalytic activities, metabolic pathways, and transcription factors. In all, 271 hQTNs were found in close vicinity of 275 candidate genes, including *Gh*_*D02G0165* which had two hQTNs associated with BN and PH; *Gh*_*D12G1396* and *Gh_A021302* each harboring two hQTNs and all four of them associated with LP; the rest of the traits were found associated with one hQTN each. The maximum number of associations between genes and traits were detected for LP, at 16, followed by MIC and FUI with 12 and 10 associations, respectively (Table 1). Of 64 putative candidate genes, 54 were considered as potential candidate genes related to the heterosis of the studied traits. Figure 10 shows the GWAS summary of the MIC-associated hQTN, hqMICD09_43629201_C, found on chromosome D09 that was contributed by the male parent A971Bt. This hQTN was found in association with all the types of heterosis as well as trait phenotypes, and it was expressed in cotton’s fiber, ovule, and different plant tissues.

**TABLE 1:** List of 64 candidate genes related to fiber quality and yield related traits with their details about biological function (GO items) annotations.

Trait	QTN/hQTN	Gene ID	Name	Start	Stop	Length (bp)	Direction	Function Description	Function annotation
BN	hqBND02_ 1523160_D	Gh_D02G0165	ARA, ARA-1, ATRAB11D, ATRABA5E, RABA5E	1520207	1522172	1965	−	RAA5E_ARATH Ras-related protein RABA5e OS = *Arabidopsis thaliana* GN = RABA5E PE = 2 SV = 1	RAB GTPase homolog A5E
BN	hqBND06_ 182732_E	Gh_D06G0024	GSO1	183401	184660	1259	−	RLP12_ARATH Receptor-like protein 12 OS = *Arabidopsis thaliana* GN = RLP12 PE = 2 SV = 2	Leucine-rich repeat transmembrane protein kinase
BN	hqBND13_ 8663474_C	Gh_D13G0621	EIF4A-III	8654254	8657278	3024	−	RH2_ORYSJ DEAD-box ATP-dependent RNA helicase 2 OS = *Oryza sativa* subsp. japonica GN = Os01g0639100 PE = 2 SV = 1	Eukaryotic initiation factor 4A-III
BW	qBWA08_ 931754_E	Gh_A08G0110	–	925654	939192	13538	−	KC1_TOXGO Casein kinase I OS = *Toxoplasma gondii* PE = 2 SV = 1	Protein kinase family protein
BW	hqBWD08_ 17717836_C	Gh_D08G0894	AIL5, CHO1, EMK	17697130	17699976	2846	−	AIL5_ARATH AP2-like ethylene-responsive transcription factor AIL5 OS = *Arabidopsis thaliana* GN = AIL5 PE = 2 SV = 2	AINTEGUMENTA-like 5
FE	hqFEA10_ 64287322_D	Gh_A10G1233	–	64261579	64264117	2538	−	FRS3_ARATH Protein FAR1-RELATED SEQUENCE 3 OS = *Arabidopsis thaliana* GN = FRS3 PE = 2 SV = 2	Far-red impaired responsive (FAR1) family protein
FE	hqFEA11_ 18660123_E	Gh_A11G1405	–	18663333	18667100	3767	−	0	Protein of unknown function (DUF1664)
FE	hqFED02_ 37337297_E	Gh_D02G1201	FHY3	37310027	37311060	1033	−	FHY3_ARATH Protein FAR-RED ELONGATED HYPOCOTYL 3 OS = *Arabidopsis thaliana* GN = FHY3 PE = 1 SV = 1	Far-red elongated hypocotyls 3
FE	hqFED09_ 42469217_C	Gh_D09G1491	ATRAB8, RAB8	42459487	42472061	12574	+	RAE1A_ARATH Ras-related protein RABE1a OS = *Arabidopsis thaliana* GN = RABE1A PE = 1 SV = 1	RAB GTPase homolog 8
FE	hqFED10_ 23461795_E	Gh_D10G1283	ATX2	23432267	23444107	11840	−	ATX2_ARATH Histone-lysine *N*-methyltransferase ATX2 OS = *Arabidopsis thaliana* GN = ATX2 PE = 2 SV = 1	Trithorax-like protein 2
FE	hqFED13_ 49321521_D	Gh_D13G1618	ATGSTU19, GST8, GSTU19	49327339	49328744	1405	+	GSTX4_TOBAC Probable glutathione *S*-transferase OS = *Nicotiana tabacum* PE = 2 SV = 1	Glutathione S-transferase TAU 19
FL	hqFLA11_ 45333662_A	Gh_A11G1858	OST1, SNRK2-6, SRK2E, SNRK2.6, P44, ATOST1	45306099	45308154	2055	+	SAPK1_ORYSJ Serine/threonine-protein kinase SAPK1 OS = *Oryza sativa* subsp. japonica GN = SAPK1 PE = 1 SV = 1	Protein kinase superfamily protein
FS	hqFSA01_ 13635803_E	Gh_A01G0714	FLA1	13649630	13651601	1971	+	FLA1_ARATH Fasciclin-like arabinogalactan protein 1 OS = *Arabidopsis thaliana* GN = FLA1 PE = 1 SV = 1	FASCICLIN-like arabinogalactan 1
FS	hqFSA01_ 83601604_A	Gh_A01G1348	–	83638071	83646061	7990	−	YAB4_ARATH Axial regulator YABBY 4 OS = *Arabidopsis thaliana* GN = YAB4 PE = 1 SV = 2	Plant-specific transcription factor YABBY family protein
FS	hqFSA04_ 48361719_A	Gh_A04G0705	ATPHAN, AS1, ATMYB91, MYB91	48314343	48315413	1070	−	AS1_ARATH Transcription factor AS1 OS = *Arabidopsis thaliana* GN = AS1 PE = 1 SV = 1	Myb-like HTH transcriptional regulator family protein
FS	hqFSA05_ 30254202_A	Gh_A05G2423	AtPP2-A12, PP2-A12	30397232	30398615	1383	−	P2A12_ARATH F-box protein PP2-A12 OS = *Arabidopsis thaliana* GN = P2A12 PE = 2 SV = 1	Phloem protein 2-A12
FS	hqFSD05_ 61238929_A	Gh_D05G3669	TIM50, emb1860	61253153	61256280	3127	−	TIM50_ARATH Mitochondrial import inner membrane translocase subunit TIM50 OS = *Arabidopsis thaliana* GN = TIM50 PE = 1 SV = 1	Haloacid dehalogenase-like hydrolase (HAD) superfamily protein
FS	hqFSD12_ 51753728_C	Gh_D12G1895	–	51753693	51760260	6567	+	MORC4_MOUSE MORC family CW-type zinc finger protein 4 OS = *Mus musculus* GN = Morc4 PE = 2 SV = 2	Histidine kinase-, DNA gyrase B-, and HSP90-like ATPase family protein
FU	hqFUA01_ 7658880_E	Gh_A01G0481	TT7, CYP75B1, D501	7653246	7654296	1050	−	C71A3_SOLME Cytochrome P450 71A3 (Fragment) OS = *Solanum melongena* GN = CYP71A3 PE = 2 SV = 1	Cytochrome P450 superfamily protein
FU	hqFUA07_ 42751600_D	Gh_A07G1461	PKT3, PED1, KAT2	42762126	42764782	2656	+	THIK2_ARATH 3-ketoacyl-CoA thiolase 2, peroxisomal OS = *Arabidopsis thaliana* GN = PED1 PE = 1 SV = 2	Peroxisomal 3-ketoacyl-CoA thiolase 3
FU	hqFUA11_ 4557423_C	Gh_A11G0474	OST1, SNRK2-6, SRK2E, SNRK2.6, P44, ATOST1	4550580	4552694	2114	+	SAPK2_ORYSI Serine/threonine-protein kinase SAPK2 OS = *Oryza sativa* subsp. indica GN = SAPK2 PE = 2 SV = 2	Protein kinase superfamily protein
FU	hqFUA11_ 4557423_C	Gh_A11G0475	AtRABA4a, RABA4a	4556537	4558130	1593	−	RB11A_LOTJA Ras-related protein Rab11A OS = *Lotus japonicus* GN = RAB11A PE = 2 SV = 1	RAB GTPase homolog A4A
FU	hqFUA12_ 3005239_D	Gh_A12G0200	ATPEN2, PEN2	2997737	3013352	15615	−	RD21A_ARATH Cysteine proteinase RD21a OS = *Arabidopsis thaliana* GN = RD21A PE = 1 SV = 1	PTEN 2
FU	hqFUA13_ 35371010_E	Gh_A13G0792	–	35286057	35287535	1478	−	FDL1_ARATH F-box/FBD/LRR-repeat protein At1g13570 OS = *Arabidopsis thaliana* GN = At1g13570 PE = 2 SV = 1	F-box/RNI-like superfamily protein
FU	hqFUD07_ 30035840_E	Gh_D07G1581	–	30009941	30010888	947	+	0	0
FU	hqFUD11_ 47125178_D	Gh_D11G2391	ATO	47096352	47101439	5087	−	SF3A3_HUMAN Splicing factor 3A subunit 3 OS = *Homo sapiens* GN = SF3A3 PE = 1 SV = 1	Splicing factor-related
FUI	hqFUIA01_ 38777600_E	Gh_A01G1066	–	38765672	38767468	1796	−	0	Protein of unknown function (DUF668)
FUI	hqFUIA03_ 6721843_D	Gh_A03G0366	SCL14, ATGRAS2, GRAS2	6718533	6720452	1919	+	SCL33_ARATH Scarecrow-like protein 33 OS = *Arabidopsis thaliana* GN = SCL33 PE = 3 SV = 1	SCARECROW-like 14
FUI	qFUIA04_ 2436958_A	Gh_A04G0150	–	2429276	2432309	3033	−	Y5162_ARATH Uncharacterized protein At5g41620 OS = *Arabidopsis thaliana* GN = At5g41620 PE = 1 SV = 2	0
FUI	hqFUIA07_ 72361363_C	Gh_A07G1773	–	72335561	72338459	2898	−	ACOT9_MOUSE Acyl-coenzyme A thioesterase 9, mitochondrial OS = *Mus musculus* GN = Acot9 PE = 1 SV = 1	Thioesterase/thiol ester dehydrase-isomerase superfamily protein
FUI	hqFUIA09_ 4569432_E	Gh_A09G0172	–	4552893	4556944	4051	−	PUR6_VIGAC Phosphoribosylaminoimidazole carboxylase, chloroplastic (Fragment) OS = *Vigna aconitifolia* GN = PURKE PE = 2 SV = 1	Phosphoribosylaminoimidazole carboxylase, putative/AIR carboxylase, putative
FUI	qFUIA12_ 86958433_A	Gh_A12G2444	–	86955300	86955536	236	+	0	0
FUI	qFUIA12_ 86958433_A	Gh_A12G2445	KT2, ATKT2, SHY3, KUP2, ATKUP2, TRK2	86956856	86960376	3520	−	POT2_ARATH Potassium transporter 2 OS = *Arabidopsis thaliana* GN = POT2 PE = 1 SV = 2	Potassium transporter 2
FUI	qFUID01_ 3569307_D	Gh_D01G0333	–	3561218	3590579	29361	+	DRL28_ARATH Probable disease resistance protein At4g27220 OS = *Arabidopsis thaliana* GN = At4g27220 PE = 2 SV = 1	NB-ARC domain-containing disease resistance protein
FUI	hqFUID06_ 20424401_A	Gh_D06G0982	TIP4;1	20390073	20393472	3399	−	TIP41_ARATH Aquaporin TIP4-1 OS = *Arabidopsis thaliana* GN = TIP4-1 PE = 2 SV = 1	Tonoplast intrinsic protein 4;1
LP	qLPA02_ 75700481_D	Gh_A02G0517	AAE7, ACN1	7668368	7749288	80920	−	AEE7_ARATH Acetate/butyrate–CoA ligase AAE7, peroxisomal OS = *Arabidopsis thaliana* GN = AAE7 PE = 1 SV = 1	Acyl-activating enzyme 7
LP	qLPA02_ 75700481_D	Gh_A02G1302	–	75725680	75725946	266	−	5GT_VERHY Anthocyanidin 3-O-glucoside 5-O-glucosyltransferase OS = *Verbena hybrida* GN = HGT8 PE = 2 SV = 1	UDP-glucosyltransferase 75B1
LP	hqLPA05_ 3272852_E	Gh_A05G0285	GPA1, GP ALPHA 1, ATGPA1	3267161	3270331	3170	+	GPA1_LUPLU Guanine nucleotide-binding protein alpha-1 subunit OS = *Lupinus luteus* GN = GPA1 PE = 2 SV = 1	G protein alpha subunit 1
LP	hqLPA05_ 3272852_E	Gh_A05G0286	–	3273106	3273747	641	+	0	0
LP	hqLPA13_ 13637771_E	Gh_A13G0580	–	13628944	13630380	1436	−	FBK8_ARATH F-box/kelch-repeat protein At1g22040 OS = *Arabidopsis thaliana* GN = At1g22040 PE = 2 SV = 1	Galactose oxidase/kelch repeat superfamily protein
LP	hqLPA13_ 35727868_C	Gh_A13G0793	–	35719907	35724367	4460	−	UCKC_DICDI Uridine-cytidine kinase C OS = *Dictyostelium discoideum* GN = udkC PE = 3 SV = 1	Phosphoribulokinase/Uridine kinase family
LP	hqLPD01_ 5332116_D	Gh_D01G0448	CUC2, ANAC098, ATCUC2	5343860	5345867	2007	+	NAC98_ARATH Protein CUP-SHAPED COTYLEDON 2 OS = *Arabidopsis thaliana* GN = NAC098 PE = 1 SV = 1	NAC (No Apical Meristem) domain transcriptional regulator superfamily protein
LP	hqLPD03_ 35830462_A	Gh_D03G1066	–	35823773	35825722	1949	−	0	0
LP	hqLPD03_ 35830462_A	Gh_D03G1067	–	35834718	35835578	860	+	BICC1_MOUSE Protein bicaudal C homolog 1 OS = *Mus musculus* GN = Bicc1 PE = 2 SV = 1	Sterile alpha motif (SAM) domain-containing protein
LP	hqLPD07_ 26600866_E	Gh_D07G1500	–	26616541	26618053	1512	+	SETH3_ARATH Probable arabinose 5-phosphate isomerase OS = *Arabidopsis thaliana* GN = SETH3 PE = 2 SV = 1	Sugar isomerase (SIS) family protein
LP	hqLPD07_ 31568560_AD	Gh_D07G1617	–	31577220	31577585	365	−	0	0
LP	qLPD10_ 10866370_ E	Gh_D10G0861	PIP3	10850015	10851179	1164	−	PIP27_ARATH Aquaporin PIP2-7 OS = *Arabidopsis thaliana* GN = PIP2-7 PE = 1 SV = 2	Plasma membrane intrinsic protein 3
LP	hqLPD12_ 43066746_CDE	Gh_D12G1396	–	43057670	43058647	977	+	0	Protein of unknown function (DUF506)
LP	hqLPD12_ 44140224_E	Gh_D12G1432	–	44129272	44129901	629	−	0	0
MIC	hqMICA02_ 7300875_C	Gh_A02G0495	–	7292521	7294588	2067	+	P2C34_ARATH Probable protein phosphatase 2C 34 OS = *Arabidopsis thaliana* GN = At3g05640 PE = 2 SV = 1	Protein phosphatase 2C family protein
MIC	hqMICA03_ 5930639_D	Gh_A03G0332	HTB4	5931201	5931644	443	+	H2B_GOSHI Histone H2B OS = *Gossypium hirsutum* GN = HIS2B PE = 2 SV = 3	Histone superfamily protein
MIC	hqMICA03_ 95492151_D	Gh_A03G1505	CPK6, ATCDPK3, ATCPK6	95507304	95510335	3031	−	CDPK4_SOLTU Calcium-dependent protein kinase 4 OS = *Solanum tuberosum* GN = CPK4 PE = 2 SV = 1	Calcium-dependent protein kinase family protein
MIC	hqMICA07_ 16344257_D	Gh_A07G0911	TOC75-III, MAR1	16336770	16340890	4120	−	TC753_ARATH Protein TOC75-3, chloroplastic OS = *Arabidopsis thaliana* GN = TOC75-3 PE = 1 SV = 1	Translocon at the outer envelope membrane of chloroplasts 75-III
MIC	hqMICD02_ 57281334_A	Gh_D02G1668	–	57265701	57267586	1885	−	RZP23_ORYSJ Serine/arginine-rich splicing factor RSZ23 OS = *Oryza sativa* subsp. japonica GN = RSZ23 PE = 2 SV = 1	Serine/arginine-rich 22
MIC	hqMICD05_ 8772003_D	Gh_D05G1039	MMT	8767120	8777841	10721	+	MMT1_ARATH Methionine *S*-methyltransferase OS = *Arabidopsis thaliana* GN = MMT1 PE = 2 SV = 1	Methionine *S*-methyltransferase
MIC	hqMICD06_ 37879191_D	Gh_D06G1287	–	37856300	37859354	3054	−	CX5B2_ARATH Cytochrome c oxidase subunit 5b-2, mitochondrial OS = *Arabidopsis thaliana* GN = COX5B-2 PE = 2 SV = 1	Rubredoxin-like superfamily protein
MIC	hqMICD09_ 43629201_C	Gh_D09G1604	PDE318	43620944	43628324	7380	−	NOG1_MOUSE Nucleolar GTP-binding protein 1 OS = *Mus musculus* GN = Gtpbp4 PE = 2 SV = 3	P-loop containing nucleoside triphosphate hydrolases superfamily protein
MIC	hqMICD09_ 43679359_C	Gh_D09G1610	–	43674575	43681355	6780	−	0	BAH domain;TFIIS helical bundle-like domain
MIC	qMICD11_ 59408001_D	Gh_D11G2909	–	59407946	59409678	1732	−	0	Uncharacterised conserved protein (UCP012943)
MIC	hqMICD12_ 45767769_E	Gh_D12G1518	HMGA	45767190	45767829	639	+	HMGYA_SOYBN HMG-Y-related protein A OS = *Glycine max* PE = 2 SV = 1	High mobility group A
MIC	hqMICD13_ 39150324_D	Gh_D13G1274	–	39197782	39198346	564	+	GOT1A_BOVIN Vesicle transport protein GOT1A OS = *Bos taurus* GN = GOLT1A PE = 2 SV = 1	Got1/Sft2-like vescicle transport protein family
PH	hqPHA11_ 88756735_C	Gh_A11G2657	–	88760230	88762677	2447	−	PP348_ARATH Pentatricopeptide repeat-containing protein At4g33990 OS = *Arabidopsis thaliana* GN = EMB2758 PE = 3 SV = 2	Pentatricopeptide repeat (PPR) superfamily protein
PH	qPHD10_8389255_D	Gh_D10G0727	–	8392851	8396066	3215	−	0	SNARE-like superfamily protein
PH	hqPHD13_ 39108563_A	Gh_D13G1273	SUC2, SUT1, ATSUC2	39078454	39081595	3141	−	SUC2_ARATH Sucrose transport protein SUC2 OS = *Arabidopsis thaliana* GN = SUC2 PE = 1 SV = 2	Sucrose-proton symporter 2

## Discussion

In conventional plant breeding, a huge number of hybrid crosses are screened to glean genotypes exhibiting ideal performance traits. However, only a few tested hybrid crosses are considered worthwhile for use as hybrid varieties. Once the heterotic loci or actual causative heterotic genes are identified with certainty, the genotypes are more likely to get scrutinized. The genotypes harboring key loci can be identified through whole-genome assembly of parental lines, by narrowing down directly those potential combinations conferring robust performances. This study is a perfect integration of both conventional and modern techniques for hybrid crosses generation which can be done quickly and with greater predictive ability. Globally, an enormous body of systematic surveys on heterosis has since accumulated. Phenological attributes have been investigated for hybrid vigor in many crops, such as grain amaranths (*Amaranthus cruentus*, *Amaranthus hypochondriacus*) (Lehmann et al., 1991), maize (Parentoni et al., 2001; Betrán et al., 2003), tomato (*Lycopersicon esculentum*) (Makesh, 2002), and rice (*Oryza sativa*) (Verma et al., 2002). The number of genotypes analyzed in those works is comparable to that accessed in our survey on cotton. In our study, we analyzed 284 female lines, four testers and their subsequent hybrid progenies across a wide spectra of environments. Hence, heterosis also ranged widely, with higher values for agronomic traits but lower values for fiber quality traits, due to the presence of many individuals with varying higher and lower phenotypic values than their parents. Because the genotype of a hybrid is obtained after the combination of both its parental genotypes, the overdominance hypothesis postulates the heterozygosity of individual loci is consequential for the superior performance. Our finding of a higher heterosis trend in agronomic traits than fiber quality traits is consistent with previous findings and can prove beneficial for cotton breeding.

Due to the scarcity of available genetic divergence in the founder parents of Cotton World stock, global climatic changes are continuously posing threats to Upland Cotton crops with respect to progress in breeding and their survival. Thus, it is imperative that we explore potential genetic diversity that might have been eroded from cultivated cotton collections. Population structure within the collection of accessions is considered crucial for explaining heterogeneity. Chinese cotton production as well as cotton breeding programs are largely based on the introduction of germplasms since long (Chen and Du, 2006). However, improved cultivated species in the last two decades have population structures with a reasonable extent of heritability. Accessions used as parents were clustered into three distinct groups on the basis of genotypic data. We identified three major subpopulations in our experimental stock of F_1_ sets, which formed at earlier stages of the cotton breeding period and were not affected by geographical influences of China.

In the last two decades, GWAS has been extensively utilized by researchers to map different quantitative traits in plants and this achievement is considered a complex milestone (Ingvarsson and Street, 2011). It is thought the power of GWAS usually depends on four distinct factors: availability of rich genetic diversity, credibility in acquisition of phenotypic data, density of markers and use of adequate statistical methods. The current collection of *G. hirsutum* accessions used as parents exhibited reasonable amounts of phenotypic and genotypic diversity. It offers a highly efficient way to mine heterosis-related loci by high-resolution GWAS in plants. Through GWAS, the relationships among significantly associated hQTNs to fiber quality as well as other agronomic traits, and the annotation of putative genes containing these hQTNs, were examined here in depth.

The identification of hQTNs using five different types of heterosis, trait performance, SCA and four F_1_ sets is another noteworthy feature of this study. In this way, the loci controlling heterosis of different traits could be separated from those concerned with trait performance in earlier studies. We distinguished 19 highly stable hQTNs for LP, BW, FL, FS and MIC traits based on their detection from five heterosis types and/or four F_1_ sets across multiple differing environments. These stable heterotic loci could be used in the future to assist in Upland Cotton breeding via MAS applications. The remaining significant hQTNs that were found related to other traits could also prove useful in cotton breeding programs. Moreover, a reasonable number of identified SNPs from the F_1_ sets and trait phenotypes overlapped with those detected from the heterosis types. These findings revealed that both heterosis and trait performance were not independently controlled by different loci, which agrees with a recent study on upland cotton (Li et al., 2018). Conversely, in rice, (Hua et al., 2003) reported them as being independently controlled by different sets of loci.

Boll weight was identified in relation with *Gh*_*D08G0894*, which encodes an ethylene-responsive transcription factor detected earlier in Arabidopsis (Nole-Wilson et al., 2005) and later in cotton (Qin et al., 2019). Ethylene is considered as a key factor in the growth of cotton fiber and in its elongation (Qin et al., 2007; Qin and Zhu, 2011); its crucial role is evident from the findings that when it occurs in excessive or insufficient amounts this negatively affects FE (Li et al., 2015). Two candidate genes, *Gh_D02G1201* and *Gh_A10G1233*, showed an association with FE-related hQTNs. Both *Gh_D02G1201* and *Gh_A10G1233* encode FAR-RED elongated proteins which are involved in light responses and FE development (Ju et al., 2019) along with the positive regulation of chlorophyll biosynthesis (Tang et al., 2012; Miao et al., 2017). *Gh_A11G1858*, which displayed an association with the hQTN related to FL, encodes a serine-threonine kinase SAPK1 protein that may play a role in the signal transduction of a hyperosmotic response markedly influencing the fiber development process (Kobayashi et al., 2004; Magwanga et al., 2018; Li et al., 2019). FU was identified with hQTNs associated with *Gh_A01G0481* and *Gh_A11G0475*; the former encodes a cytochrome P450 protein which may have a role in the maturation or aging of tissues (Ju et al., 2019), while the latter encodes the Ras-related protein RAB11A, it detected previously in fiber development and elongation (Qin et al., 2017). The FUI trait was associated with a hQTN related to *Gh_A03G0366*, which encodes a scarecrow-like protein that acts as transcription factor and which regulates the development of vegetative and reproductive plant parts (Cenci and Rouard, 2017; Zhang et al., 2018). The LP-associated hQTN was related to *Gh_A05G0285*, a gene earlier detected in cotton for coding nucleotide binding protein responsible for fiber development (Ju et al., 2019). The MIC-directed association toward hQTNs related to *Gh*_*A03G1505*, *Gh_A03G0332*, *Gh_A02G0495*, and *Gh_D06G1287*; these reportedly encode in cotton the production of calcium-dependent proteins related respectively to the kinase family (Si et al., 2020), histones H2B (Qin et al., 2019), phosphatase 2C (Ju et al., 2019; Song et al., 2019; Shahzad et al., 2020), and cytochrome C oxidase (Zhang et al., 2019). Finally, FS was associated with a hQTN related to the *Gh_A01G1348* gene known for encoding axial regulator proteins controlling the biomass vigor of hybrid cotton (Shahzad et al., 2020).

The current study is based on the concept of genomic hybrid breeding, previously utilized in rice (Xu et al., 2014), which exploited the strategy of genome sequencing. The sequence data was then deployed to evaluate F_1_ progenies’ performance in hybrid breeding. An earlier study on rice revealed the power of SNP-directed yield estimation of F_1_ hybrids. In the current study, 298 QTNs were uncovered in association with fiber quality as well as agronomic traits. A set of 271 hQTNs were detected with 19 highly stable heterotic loci in relation with LP, BW, FL, FS, and MIC based on their detection from five evaluated types of heterosis and/or four F_1_ hybrid sets across a wide spectrum of environments. These discovered hQTNs and putative candidate genes related to HETEROSIS of quoted traits could be used further deliberately in marker-assisted breeding of forthcoming cotton hybrid breeding programs. Once the genotype-based predictions achieve relatively high levels of accuracy, the labor and time costs of hybrid breeding are greatly reduced. The reported information derived in this study is of practical and scientific significance for both cotton breeders and biologists engaged in elucidating the heterosis mechanism of fiber as it could assist in successful accomplishments in both domains.

## Data Availability Statement

The data related to RAD-sequencing have been submitted to NCBI under reference No. PRJNA353524. Other used data are provided in the form of Supplementary Material/Tables. Any other information supporting the conclusions of this article, if needed, will be made available by the authors, at appropriate request without undue reservation.

## Author Contributions

XD and JS: conceived and designed the research. JS, QW, HQ, JL, HL, JiY, ZM, and DX: managed the project. ZS, ZP, XG, MShI, MFN, MSaI, and HA: designed and performed molecular experiments in lab along with molecular data analysis. YJ, SH, JS, HQ, HL, DX, JuY, JZ, ZL, ZC, XiZ, XuZ, AH, XY, GZ, LL, HZ, BP, and LW: prepared samples and performed phenotyping in Anyang, Henan, Xinxiang, Wuhan, Jingzhou, Baoding, Changde, Shandong etc. ZS, SA, and MShI: analyzed and interpreted data and prepared figures and tables. ZS, MShI, and XD: drafted and processed the manuscript and all authors helped throughout this process and take active part in critical revisions and improvements in important intellectual contents. All authors read the manuscript critically and approved the final version of manuscript for publication. All authors agreed to be accountable for all aspects of the work in ensuring that questions related to the accuracy or integrity of any part of the work are appropriately investigated and resolved.

## Conflict of Interest

It is declared that, authors, JL, JY, ZC, and GZ were employed by “Zhongmian Seed Technologies Co., Ltd., Zhengzhou, China,” the author, HL employed by “Jing Hua Seed Industry Technologies Inc., Jingzhou, China,” the author, DX employed by “Guoxin Rural Technical Service Association, Hebei, China,” the authors, JZ as well as LL were employed by “Zhongli Company of Shandong, Shandong, China,” and the author AH employed by “Sanyi Seed Industry of Changde in Hunan Inc., Changde, China.” The remaining authors declare that the research was conducted in the absence of any commercial or financial relationships that could be construed as a potential conflict of interest.

## Footnotes

1 http://mascotton.njau.edu.cn/info/1054/1118.htm

2 http://128.32.118.212/thorfinn/realSFS/

3 https://cottonfgd.org

## Abbreviations

BN: boll number
Bt: *Bacillus thuringiensis*
Dim: dimension
DNA: deoxyribonucleic acid
F_1_: first filial generation
FE: fiber elongation
FL: upper half mean Length
FS: fiber strength
FU: fiber uniformity
FUI: fiber uniformity index
HB: heterobeltosis
HI: heterosis index
hQTN: heterotic Quantitative Trait Nucleotide
K3: competitive heterosis over check Rui za 816
K4: competitive heterosis over check Eza mian 10 hao (Tai D5)
L × T: Line into Tester mating design
LD: linkage disequilibrium
LP: lint percentage
Mb: Million base pairs
MIC: fiber micronaire
MP: Mid-Parent heterosis
PCA: principal component analysis
PH: plant height
r: Correlation
r^2^: coefficient of regression
SCA: specific combining ability
